# Social engagement and depressive symptoms in Korean older adults: The potential moderating role of employment status

**DOI:** 10.1371/journal.pone.0342299

**Published:** 2026-03-05

**Authors:** Ji-Eun Lee, Seonji Kim, Jeehye Lee, Yun-Chul Hong, Kyung-Shin Lee, Hye Sook Min

**Affiliations:** 1 Department of Human Systems Medicine, College of Medicine, Seoul National University, Seoul, South Korea; 2 Department of Biomedical Systems Informatics, Yonsei University College of Medicine, Seoul, South Korea; 3 Institute for Innovation in Digital Healthcare, Yonsei University, Seoul, South Korea; 4 Department of Preventive Medicine, College of Medicine, Konkuk University, Chungju, South Korea; 5 Institute of Environmental Medicine, Seoul National University Medical Research Center, Seoul, South Korea; 6 Center for Public Healthcare Policy, National Medical Center, Seoul, South Korea; 7 Public Healthcare Research Institute, National Medical Center, Seoul, South Korea; Ajou University School of Medicine and Graduate School of Medicine, KOREA, REPUBLIC OF

## Abstract

**Background:**

Social engagement is known to alleviate depressive symptoms among older adults, and continued employment in later life may serve as a key mechanism for sustaining social engagement. However, the impact of employment on mental health can vary depending on job quality and socioeconomic conditions. This study aims to examine the association between social engagement, employment status, and depressive symptoms among older adults in South Korea.

**Methods:**

This study utilized data from the Korea Community Health Survey conducted biennially from 2017 to 2023, excluding 2021. The analytic sample consisted of 199,205 adults aged 65 years and older. Depressive symptoms were assessed using the Patient Health Questionnaire-9. The effects of social engagement, categorized by informal and formal engagement, were analyzed in subgroups based on employment status. Associations were examined using multivariable logistic regression analysis.

**Results:**

All types of social engagement were significantly associated with depressive symptoms, with the strongest associations observed for contact with friends and participation in leisure or recreational activities. Employment status served as effect modifier of these relationships, with employed older adults generally showing a lower likelihood of depressive symptoms compared to their unemployed counterparts. The negative association with employment was particularly pronounced in the domains of friend contact and all types of formal social engagement.

**Discussion:**

Our findings highlight the importance of promoting formal social engagement as a potential intervention strategy for preventing depression among older adults and emphasize the need to consider appropriate forms of employment as part of this strategy.

## 1. Introduction

According to the Global Burden of Disease Study 2021, the age-standardized point prevalence of depressive disorder among adults aged 60 and older was 6.2% worldwide, the highest among all mental disorders [1]. Depression among older adults leads to significant health consequences, including reduced quality of life, cognitive decline, and increased mortality, all of which may have broader societal implications [2,3].

South Korea is one of the most rapidly aging societies in the world, with adults aged 65 and older accounting for 19.2% of the total population as of 2024 [4]. According to recent national surveys using the Short Form of the Geriatric Depression Scale (SGDS), 11.3% of older adults exhibit depressive symptoms when a cutoff score of 8 or higher is applied [5]. Older adults in Korea have been reported to show a higher prevalence of depression compared to other age groups, a trend attributed to rapid socioeconomic changes, shifts in family structure, and weakened social support, and reductions of social engagement. [6,7]. Among these, social engagement has been recognized as a modifiable factor and a critical target for preventive interventions [8]. This emphasis on social engagement is grounded in activity theory, which posits that successful aging involves maintaining social roles, activities, and a sense of purpose established in midlife. From this perspective, continued participation in social activities is considered as an important component of successful aging and has been consistently associated with better mental health outcomes in later life [9,10].

Many studies have increasingly shown that participation in social activities is positively associated with reduced depressive symptoms among older adults. A multinational study of European adults aged 50 and older found that social participation was generally linked to improvements in depressive symptoms, although the direction and strength of this association varied by the type of activity [11]. Similarly, studies in East Asian populations have consistently shown that sustained participation, higher frequency of social interactions, and involvement in a diverse range of activities are associated with a lower risk of depressive symptoms in later life [12–15].

Withdrawal from the workforce in later life represents a major transition away from economic activity and social roles, and is known to considerably affect levels of social engagement. Even when controlling for the aging process, non-employment in later life has been closely associated with reductions in the size of social networks and peripheral relationships [16]. In South Korea, retired older adults have been reported to experience a gradual decline in the frequency of meeting friends and a sharp decrease in participation in social gatherings compared to their working peers [17]. These changes suggest that retirement may contribute to an increased risk of depression. However, the mental health effects of continued employment in later life remain complex, and findings across studies are inconsistent. While some previous studies have observed an inverse association between employment and depression risk, the strength and direction of this relationship appear to vary by job type, working hours, and age [18–20]. A recent meta-analysis indicated that the transition to retirement is associated with a higher risk of depression, although this relationship varies by the type of retirement and national contexts [21].

In South Korea, late-life employment and quality of life remain critical areas in need of significant improvement. Although the labor force participation rate among adults aged 65 and older reached 38.3% in 2023 [22], the elderly poverty rate remained alarmingly high at 39.7% in 2022, substantially exceeding rates in other countries [23]. This disparity is likely attributable to the insufficiency of the public pension system and the predominance of low-wage, insecure, and physically demanding jobs among older workers [24]. Building on this theoretical and empirical foundation, the present study aims to examine how different types of social engagement are associated with depressive symptoms among older adults in South Korea, and whether these associations vary by employment status. Using data from the 2021 Korea Community Health Survey (KCHS), we explore the moderating role of employment in the relationship between social engagement and mental health in later life.

## 2. Methods

### 2.1. Study design and sample

This study is a cross-sectional analysis based on the data from the KCHS provided by the Korea Centers for Disease Control and Prevention (KDCA), a nationwide survey conducted since 2008 to produce health statistics for developing community health plans. The KCHS targets community-dwelling adults aged 19 and older and employs a stratified multistage sampling method. Data are collected through face-to-face interviews conducted by trained interviewers. All data were fully anonymized with personal identifiers removed prior to public release. Detailed information on the sample design and contents of the KCHS has been previously reported [25]. To ensure adequate sample size, increase the accuracy and reliability of the estimates, and improve generalizability, we combined data from 2017, 2019, and 2023 survey waves. Although the KCHS routinely includes relevant variables, data from 2021 were excluded because items related to social engagement were omitted during the COVID-19 pandemic.

**Fig 1:**
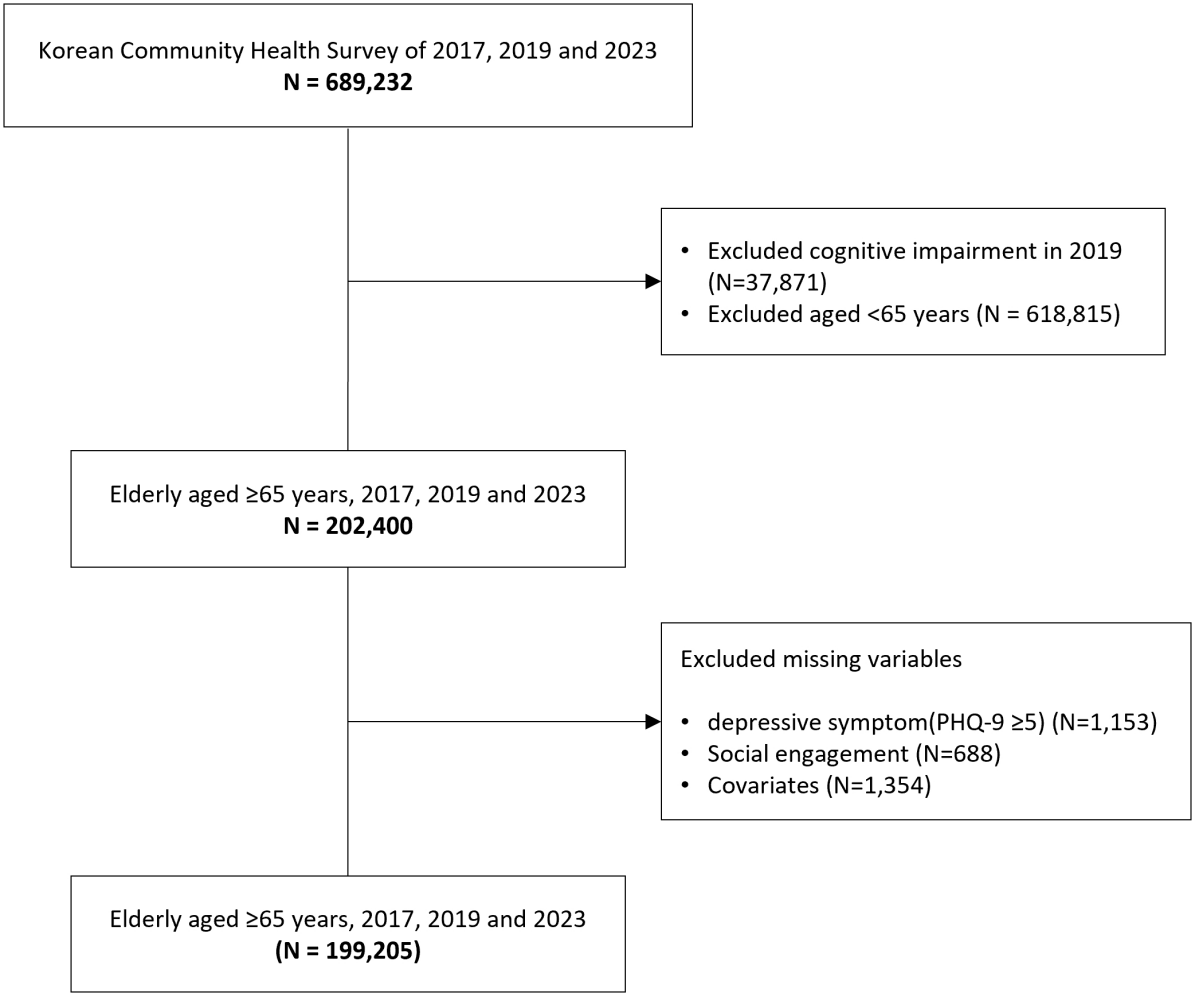
Flow chart of study population selection.

The initial dataset included 689,232 individuals. We first excluded 37,871 respondents from the 2019 survey who reported cognitive impairments that could compromise the reliability of responses to depressive symptom items. Cognitive impairment was determined based on a ‘yes’ response to the question, ‘In the past year, have you experienced mental confusion or memory loss more frequently or more severely than before?’. This item was included only in the 2019 survey and was not available in the 2017 and 2023 surveys. Accordingly, a sensitivity analysis, described in the following section, was conducted to address this limitation. We then restricted the sample to individuals aged 65 and older and excluded an additional 3,195 respondents with missing data. The final analytic sample consisted of 199,205 older adults (Fig 1). This study was approved by the Institutional Review Board of the National Medical Center (approval number: NMC-2023-03-037) and data were obtained for study purposes on 08 May 2023.

### 2.2. Measurement of social engagement

In this study, social engagement was assessed by categorizing specific types of social activities and quantifying their frequency. Consistent with previous research, interactions with friends, neighbors, and relatives were classified as informal social engagement, while participation in religious activities, volunteering, social gatherings, leisure/recreational activities, and charity/volunteer activities was categorized as formal social engagement [26–28]. Informal engagement was measured using the following questions: 1) ‘How often do you meet or communicate with the relative (including family members) with whom you have the most contact?’ 2) ‘How often do you meet or communicate with the neighbor you have the most contact with?’ 3) ‘How often do you meet or communicate with the friend (excluding neighbors) you have the most contact with?’ Responses were recorded as: ‘less than once a month’, ‘once a month’, ‘2–3 times a month’, ‘once a week’, ‘2–3 times a week’, or ‘4 or more times a week’. Formal engagement was assessed with the following yes/no items: 4) ‘Do you regularly participate in religious activities at least once a month?’ 5) ‘Do you regularly participate in social gatherings (such as clan gathering, alumni associations, senior citizen centers, neighborhood associations, etc.) at least once a month?’ 6) ‘Do you regularly participate in leisure/sports activities at least once a month?’ 7) ‘Do you regularly participate in charity/voluntary activities at least once a month?’ Respondents who reported informal engagement as ‘less than once a month’ or formal engagement as ‘yes were categorized separately for statistical analysis.

### 2.3. Assessment of depressive symptoms

Depressive symptoms were assessed using the Korean version of the Patient Health Questionnaire-9 (PHQ-9) [29]. The PHQ-9 is widely used as a screening tool for depressive symptoms in the general population and has been validated for use in the Korean population [30]. The PHQ-9 consists of nine items that evaluate the frequency of depressive symptoms over the past two weeks [31]. Based on recent East Asian studies recommending a cutoff score of 5 for identifying major depression in older adults [28,32], participants were categorized as having depressive symptoms or not based on a PHQ-9 score of ≥ 5. In this study, the term “depressive symptoms” therefore refers to participants scoring at or above this cutoff (PHQ-9 ≥ 5). The Cronbach’s α coefficient for the PHQ-9 in this study was 0.84.

### 2.4. Employment status

Employment status was defined in accordance with the International Labor Organization (ILO) standards [33]. It was measured using responses to the following question: ‘In the past week, have you worked for more than one hour for pay, or worked as an unpaid family worker for more than 18 hours?’ Individuals who answered ‘yes were considered employed.

### 2.5. Covariates

Demographic covariates included age (65–74, 75–84, or ≥ 85 years), sex (male or female), survey year (2017, 2019 and 2023), marital status (married or other), educational level (middle school or below, high school or above), living alone (yes or no), monthly household income in Korean won (KRW) (< 1 million, 1–2.99 million, 3 million or more), residential area (metropolitan cities or provinces), diabetes (yes or no), and hypertension (yes or no). Marital status labeled as other included individuals who were never married, separated, divorced, or widowed. Health behavior covariates included current smoking (yes or no), current drinking (yes or no), and moderate-intensity physical activity (yes or no). Current status of smoking and drinking was defined based on engagement in the behavior at least once per month over the past year. Moderate-intensity physical activity was defined as engaging in either vigorous physical activity for more than 20 minutes on at least three days per week or moderate activity for more than 30 minutes on at least five days per week in the past week. Lastly, self-rated health was used as variables related to quality of life, which was dichotomized as good (very good or good) and poor (fair, poor, or very poor).

### 2.6. Statistical analysis

Descriptive statistics were used to summarize participant characteristics according to the presence of depressive symptoms, and group differences were assessed using the Rao-Scott chi-square test. Multivariable logistic regression analysis was performed to examine the association between variables related to social engagement and depressive symptoms (Model 1 and Model 2), and the effect modifier of employment status was evaluated by including an interaction term for each social engagement variable x employment status (Model 3). Results are presented as adjusted odds ratios (aORs) with 95% confidence intervals (CIs). In addition, we conducted combined stratification by employment status (employed vs. unemployed) and income level (low, middle, and high), creating six distinct subgroups to estimate stratum-specific aOR with adjustment.

To assess the robustness of our findings, we performed fives sensitivity analyses: (1) a stratified analysis by age group (young-old: 65–74 years; old-old: ≥ 75 years) to examine potential differences in social engagement and depressive symptoms across age groups, (2) a comparison between the pooled 2017–2019 dataset and the 2023 dataset, (3) an analysis using an alternative three-category classification of informal social engagement (<1/month, 1–3/month, and ≥1/week), and (4) a stratified analysis by employment status to clarify the interaction effect between employment and social engagement on depressive symptoms. (5) Although we used a cut-off of ≥5 based on evidence of its validity in Asian populations, we conducted a sensitivity analysis using the more widely adopted cut-off of ≥10 for moderate to severe depression. All statistical analyses were performed using SAS software (version 9.4; SAS Institute Inc., Cary, NC, USA) and R software version 4.3.0. Statistical significance was set at p < 0.05, with a 95% CI.

## 3. Results

### 3.1. Descriptive statistics

Among the 199,205 participants, 34,982 (18.47%) scored above the cut-score for clinically significant depressive symptoms (PHQ-9 score ≥ 5) (Table 1). The rate of depressive symptoms (PHQ-9 score ≥ 5) was higher among participants with middle school or lower education (21.48%) than those with high education (13.03%), and among those with a household income of less than 1 million (27.15%) compared with those with a household income of 3 million or more (13.12%), showing a clear socioeconomic disparity. Those living alone (25.86%) had a higher rate of depressive symptoms (PHQ-9 score ≥ 5) than those who were living with others (16.51%).

**Table 1:** Descriptive statistics and related factors to depressive symptoms (PHQ-9 score ≥ 5) in the study population (n, weighted %).

Participant characteristics	Categories	Total (N = 199,205)	Depressive symptoms (PHQ-9 ≥ 5)		
			No (N = 164,223)	Yes (N = 34,982)	*Rao-Scott* *X* ^*2*^ *(p)*
Age group	65-74	108,656 (59.10)	93,692 (85.16)	14,964 (14.84)	1487.74 (<0.0001)
	75-84	74,538 (34.26)	58,943 (77.64)	15,595 (22.36)	
	≥ 85	16,011 (6.64)	11,588 (67.33)	4,423 (30.67)	
Sex	Male	84,659 (45.18)	74,056 (86.65)	10,603 (13.35)	1440.18 (<0.0001)
	Female	114,546 (54.82)	90,167 (77.31)	24,379 (22.69)	
Survey year	2017	66,452 (32.31)	53,683 (79.87)	12,769 (20.13)	1010.29 (<0.0001)
	2019	51,559 (24.96)	44,582 (86.32)	6,977 (13.68)	
	2023	81,194 (42.73)	65,958 (79.99)	15,236 (20.01)	
Marital status	Married	72,196 (34.05)	55,071 (74.51)	17,125 (25.49)	1485.24 (<0.0001)
	Others^a^	127,009 (65.95)	109,152 (85,15)	17,857 (14,84)	
Education	Middle school or lower	147,598 (64.33)	118,711 (78.52)	28,887 (21.48)	871.65 (<0.0001)
	High school or higher	51,607 (35.67)	45,512 (86.97)	6,095 (13.03)	
Employment status	No	116,418 (67.58)	91,197 (78.27)	25,221 (21.73)	1487.02 (<0.0001)
	Yes	82,787 (32.42)	73,026 (88.33)	9,761 (11.67)	
Living alone	No	147,641 (79.08)	124,920 (83.49)	22,721 (16.51)	980.70 (<0.0001)
	Yes	51,564 (20.92)	39,303 (74.14)	12,261 (25.86)	
Household income (KRW)^b^	< 1 million	75,279 (28.20)	57,261 (72.85)	18,018 (27.15)	1751.83 (<0.0001)
	1-2.99 million	82,528 (42.71)	70,569 (83.62)	11,959 (16.38)	
	≥ 3 million	41,398 (29.09)	36,393 (86.88)	5,005 (13.12)	
Residence area	Metropolitan	47,779 (42.69)	38,935 (81.37)	8,844 (18.63)	0.98 (0.32)
	Province	151,426 (57.31)	125,288 (81.65)	26,138 (18.35)	
Diabetes	No	154,327 (76.82)	128,491 (82.48)	25,836 (17.52)	187.67 (<0.0001)
	Yes	44,878 (23.18)	35,732 (78.39)	9,146 (21.61)	
hypertension	No	89,169 (45.82)	75,055 (83.44)	14,114 (16.56)	196.34 (<0.0001)
	Yes	110,036 (54.18)	89,168 (79.92)	20,868 (20.08)	
Current smoking	No	180,932 (90.52)	148,931 (81.46)	32,001 (18.54)	3.50 (0.06)
	Yes	18,273 (9.48)	15,292 (82.27)	2,981 (17.73)	
Current drinking	No	143,142 (69.72)	115,006 (79.07)	28,136 (20.93)	852.19 (<0.0001)
	Yes	56,063 (30.28)	49,217 (87.20)	6,846 (12.80)	
Physical activity	No	164,013 (83.54)	133,401 (80.30)	30,612 (19.70)	481.88 (<0.0001)
	Yes	35,192 (16.46)	30,822 (87.79)	4,370 (12.21)	
Self-rated health	Poor	156,124 (76.13)	123,394 (77.51)	32,730 (22.49)	3112.79 (<0.0001)
	Good	43,081 (23.87)	40,829 (94.35)	2,252 (5.65)	

^a^ Never married, separated, divorced, or widowed; ^**b**^ KRW 1.0 million is approximately USD 1,000.

In Table 2, 27.66% of older adults reported less than monthly contact with friends, followed by 27.54% having less than monthly contact with neighbors. Among those with insufficient informal engagement, the prevalence of depressive symptoms (PHQ-9 score ≥ 5) was highest in participants reporting less than monthly contact with friends (27.68%). For formal engagement, the rate of volunteer activities less than once a month was the highest at 93.54%, and social gathering was the lowest at 48.28%. The rates of depressive symptoms (PHQ-9 score ≥ 5) among older adults who participated in formal engagement less than once a month was as follows: social gatherings (24.44%), leisure activities (20.67%), religious activities (19.37%), and charity/volunteer activities (19.08%).

**Table 2:** Distribution of social engagement in the study population.

Type of engagement		Categories	Total (N=199,205)	Depressive symptoms (PHQ-9 ≥ 5)		
				No (N=164,223)	Yes (N=34,982)	*Rao-Scott* *X* ^*2*^ *(p)*
**Informal**	Contact with relative	≥1 / month	174,368 (85.61)	145,311 (82.57)	29,057 (17.43)	359.22 (<0.0001)
		< 1 / month	24,837 (14.39)	18,912 (75.38)	5,925 (24.62)	
	Contact with neighbor	≥1 / month	164,606 (70.46)	137,576 (83.01)	27,030 (16.99)	274.73 (<0.0001)
		< 1 / month	34,599 (27.54)	26,647 (78.00)	7,952 (22.00)	
	Contact with friend	≥1 / month	139,679 (72.34)	119,673 (85.05)	20,006 (14.95)	1961.41 (<0.0001)
		< 1 / month	59,526 (27.66)	44,550 (72.32)	14,976 (27.68)	
**Formal**	Religious activity	≥1 / month	63,263 (36.04)	52,891 (83.13)	10,372 (16.87)	84.72 (<0.0001)
		< 1 / month	135,942 (63.96)	111,332 (80.63)	24,610 (19.37)	
	Social gatherings	≥1 / month	98,828 (51.72)	86,495 (87.11)	12,333 (12.89)	2006.17 (<0.0001)
		< 1 / month	100,377 (48.28)	77,728 (75.56)	22,649 (24.44)	
	Leisure/recreational activities	≥1 / month	32,769 (21.01)	29,642 (89.83)	3,127 (10.17)	1019.82 (<0.0001)
		< 1 / month	166,436 (78.99)	134,581 (79.33)	31,855 (20.67)	
	Charity/volunteer activities	≥1 / month	11,202 (6.46)	10,216 (90.41)	986 (9.59)	299.69 (<0.0001)
		< 1 / month	188,003 (93.54)	154,007 (80.92)	33,996 (19.08)	

### 3.2. The role of employment in the relationship between social engagement and depressive symptom

Table 3 presents the results of multivariate logistic regression analyses showing that different types of social engagement were differentially associated with depressive symptoms (PHQ-9 score ≥ 5) among older adults. Results from Model 2 showed that, for all types of informal engagement, participating less than once a month was associated with higher odds of depressive symptoms compared with more frequent engagement, with the strongest association observed for contact with friends (aOR = 1.66, 95% CI [1.60–1.72]). Similarly, infrequent engagement in formal engagement was strongly associated with depressive symptoms (PHQ-9 score ≥ 5), with the highest aOR for social gatherings (aOR = 1.60, 95% CI [1.54–1.66]). Being employed consistently showed an inverse association with depressive symptoms (PHQ-9 score ≥ 5) across all social engagement, except for charity/volunteer activities in Model 2. In Model 3, statistically significant interactions were found for all variables except for contacts with relatives, being more evident in two types of formal social engagement: charity/volunteer activities (aOR = 0.63, 95% CI [0.52–0.76]) and social gatherings (aOR = 0.74, 95% CI [0.69–0.80]). These interaction effects indicated that the inverse association of employment was more pronounced among those with low levels of formal or informal social engagement (<1/month), suggesting a compensatory role of employment for socially isolated older adults.

**Table 3:** Multivariable regression models for social engagement associated with depressive symptoms (PHQ-9 score ≥ 5).

Type of engagement	Variables	Categories	aOR (95% CI)		
			Model 1	Model 2	Model 3
Informal	Contact with relative (ref: ≥ 1/ month)	< 1/ month	1.55 (1.48–1.62)*	1.44 (1.37–1.51)*	1.45 (1.38,1.53)*
	Employment status (ref: No)	Yes		0.66 (0.63–0.68)*	0.66 (0.63,0.69)*
	Contact with relative x Employment status				0.96 (0.86,1.07)
	R-square		0.0040	0.0826	0.0826
	Contact with neighbor (ref: ≥ 1/ month)	< 1/ month	1.38 (1.33–1.43)*	1.51 (1.45–1.57)*	1.54 (1.48,1.62)*
	Employment status (ref: No)	Yes		0.67 (0.64–0.70)*	0.69 (0.66,0.72)*
	Contact with neighbor x Employment status				0.89 (0.81,0.97)*
	R-square		0.0034	0.0847	0.0847
	Contact with friend (ref: ≥ 1/ month)	< 1/ month	2.18 (2.10–2.26)*	1.66 (1.60–1.72)*	1.73 (1.66,1.81)*
	Employment status (ref: No)	Yes		0.67 (0.64–0.70)*	0.72 (0.68,0.75)*
	Contact with friend x Employment status				0.78 (0.72,0.85)*
	R-square		0.0201	0.0841	0.0873
Formal	Religious activity (ref: ≥ 1/ month)	< 1/ month	1.18 (1.14–1.23)*	1.21 (1.16–1.25)*	1.23 (1.17,1.28)*
	Employment status (ref: No)	Yes		0.65 (0.62–0.68)*	0.69 (0.64,0.74)*
	Religious activity x Employment status				0.91 (0.84,0.99)*
	R-square		0.0010	0.0812	0.0813
	Social gatherings (ref: ≥ 1/ month)	< 1/ month	2.19 (2.11–2.26)*	1.57 (1.52–1.63)*	1.69 (1.62,1.77)*
	Employment status (ref: No)	Yes		0.67 (0.64–0.70)*	0.78 (0.73,0.83)*
	Social gatherings x Employment status				0.75 (0.69,0.81)*
	R-square		0.0221	0.0860	0.0865
	Leisure/recreational activities (ref: ≥ 1/ month)	< 1/ month	2.30 (2.18–2.43)*	1.50 (1.42–1.59)*	1.58 (1.48,1.69)*
	Employment status (ref: No)	Yes		0.64 (0.61–0.67)*	0.77 (0.69,0.86)*
	Leisure/recreational activities x Employment status				0.81 (0.72,0.91)*
	R-square		0.0135	0.0826	0.0827
	Charity/volunteer activities (ref: ≥ 1/ month)	< 1/ month	2.22 (2.03–2.44)*	1.47 (1.34–1.63)*	1.70 (1.51,1.92)*
	Employment status (ref: No)	Yes		0.65 (0.63–0.68)*	0.99 (0.82,1.20)
	Charity/volunteer activities x Employment status				0.64 (0.53,0.78)*
	R-square		0.0042	0.0811	0.0813

Abbreviation: aOR, adjusted odds ratio; CI, confidence interval. For interaction terms, aORs represent the ratio of odds ratios comparing the effect of social engagement on depressive symptoms between employed and non-employed groups. Model 1: unadjusted model; Model 2: adjusted for age group, sex, survey year, marital status, education, living alone, household income, residence area, diabetes, hypertension, current smoking, current drinking, moderate-intensity physical activity; Model 3: adjusted for model 2 + interactive term.

* p < 0.05.

### 3.3. Stratified analysis by employment and income subgroups

**Fig 2:**
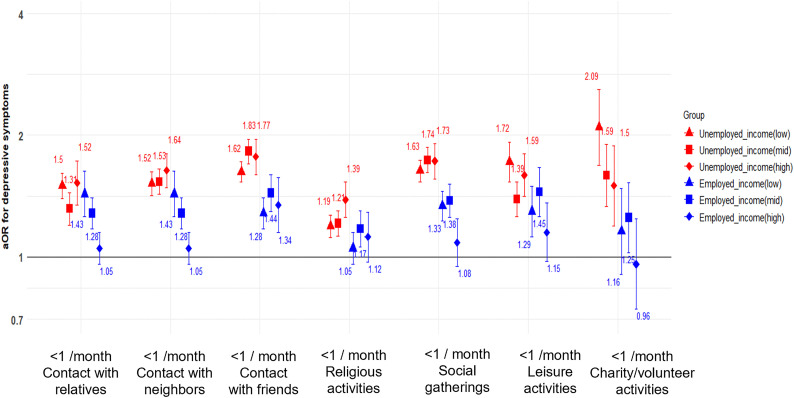
Adjusted odds ratios (aORs) with 95% confidence interval(CI)s for social engagement and depressive symptoms (PHQ-9 score≥ 5) according to income and employment status. Participants were categorized into six groups based on employment status (employed/unemployed) and income levels (low/mid/high). Red symbols represent unemployed groups and blue symbols represent employed groups. Triangle markers indicate low income, square markers indicate middle income, and diamond markers indicate high income groups. Values above 1.0 indicate increased odds of depressive symptoms compared to the reference group.

Fig 2 shows fully adjusted results for the impact of each social engagement variable on depressive symptoms (PHQ-9 score ≥ 5) by multi-stratified on income and employment status. Unemployed older adults demonstrated higher aOR for depressive symptoms (PHQ-9 score ≥ 5) across most social engagement compared to employed groups. In particular, formal engagement had a more pronounced effect on depressive symptoms (PHQ-9 score ≥ 5) than informal engagement across all income groups, with the strongest differences observed among higher income groups. This was especially evident in contact with contact with friends (aOR=1.34, 95% CI: 1.15–1.57 in employed; aOR=1.77, 95% CI: 1.60–1.96 in unemployed), social gatherings (aOR=1.09, 95% CI: 0.95–1.25 in employed; aOR=1.73, 95% CI: 1.56–1.91 in unemployed) and charity (aOR=0.96, 95% CI: 0.74–1.24 in employed; aOR=1.50, 95% CI: 1.19–1.89 in unemployed) in S1 Table.

### 3.4. Sensitivity analysis

To assess the robustness of our main findings, we conducted four sensitivity analyses. Although there were statistically significant differences in the prevalence of depressive symptoms (PHQ-9 score ≥ 5) in age groups (young-old: 14.84%; old-old: 23.71%), the association between social engagement and depressive symptoms remained largely consistent in the two groups, except for the contact with relatives and leisure activity (S2 Table). In the second sensitivity analysis, a comparison was made between two groups: data from 2017 and 2019 combined and data from 2023. As a result, a significantly stronger associations were observed for contact with relatives and contact with neighbors (S3 Table). However, no such results were observed for formal engagement, suggesting that the relationship between social engagement and depressive symptoms (PHQ-9 score ≥ 5) remained stable. In the third sensitivity analysis, the informal engagement scale was divided into three categories (< 1/ month, 1–3/ month, and ≥ 1/ week), and its association with depressive symptoms (PHQ-9 score ≥ 5) was examined (S1 Fig). The results showed that more frequent contact was associated with a lower risk of depressive symptoms, with the strongest association found among those who had contact with friends more than once a week. Fourth, the stratified analysis by employment status revealed that unemployed older adults who participated in formal or informal engagement less than once a month were at a higher risk of depressive symptoms (S2 Fig). These results support the robustness of our main findings, indicating that the associations between social engagement and depressive symptoms (PHQ-9 score ≥ 5) were consistent across different age groups and years, with only minor variations in effect sizes. Finally, the results when analyzed with a PHQ-9 score ≥ 10 were similar to those with a PHQ-9 score ≥ 5 (S4 Table).

## 4. Discussion

Our findings indicate that all types of social engagement were significantly associated with depressive symptoms (PHQ-9 score ≥ 5), with the strongest associations observed for contact with friends among informal engagement and participation in leisure or recreational activities among formal engagement. Employment status served as effect modifier of these relationships, with employed older adults generally showing a lower likelihood of depressive symptoms (PHQ-9 score ≥ 5) compared with their unemployed counterparts. Employment was more strongly associated with lower odds of depressive symptoms in contact with friends and in all types of formal social engagement.

The findings of this study are consistent with previous evidence suggesting that formal social engagement, such as participation in leisure activities and volunteering, contributes to the alleviation of depressive symptoms among older adults [11,34]. A Korean panel study similarly found that formal engagement, including religious activities, social gatherings, and volunteering, had a significant positive impact on the quality of life among older adults [35]. Another study reported that formal engagement was more effective than informal engagement in reducing depressive symptoms [26]. However, the association between formal engagement and reduced depressive symptoms has not been consistently observed across all studies. A longitudinal study in Korea highlighted the complexity of these associations, indicating that informal engagement, such as contact with friends and family, was linked to a reduction in depressive symptoms, but only among those with good baseline mental health [36]. In contrast, among formal types of engagement, religious activity was found to be associated with an increase in depressive symptoms. In a recent study, Hajek and König reported that increased participation in social activities was associated with higher psychological distress when the subjective sense of social connectedness was low, suggesting that participation alone may not be sufficient to enhance mental well-being [37]. Taken together, these findings indicate that the mental health benefits of social engagement depend not only on the type of activity but also on the individual’s initial mental health status, cultural context, and most importantly, the quality of social connectedness and emotional support. Formal engagement may enhance psychological well-being by expanding social networks and improving access to various social resources and support systems [27]. However, what may play a more decisive role in mitigating depressive symptoms is not merely the act of participation itself, but rather the sense of group cohesion and belonging that emerges through the participatory experience [38]. Further longitudinal research is needed to further explore this relationship.

Several studies reported that family-centered networks in East Asian cultures have traditionally played a positive role in supporting the physical and psychological well-being of older adults [39,40]. However, recent research, including the findings of this study, suggests a shifting role of family networks, with friendships emerging as increasingly important. Although East Asian societies have historically emphasized family-oriented social structures, rapid socioeconomic changes in South Korea led older adults to expand their social networks beyond family. In this context, friend-centered networks have become major sources of social support, and have been linked to improved mental health outcomes [41]. Similarly, a study from Japan reported that a lack of contact with friends and insufficient formal social engagement had a greater adverse impact on health outcomes than the absence of family-related interactions [42]. A study of Chinese older adults who experienced negative events found that family ties were not significant, but friendship ties significantly moderated depressive symptoms [43]. These findings suggest that friendships may play an increasingly critical role in promoting the mental health of older adults, potentially surpassing the influence of traditional family ties.

Our finding showed that employment serves as effect modifier in association between social engagement and depressive symptoms (PHQ-9 score ≥ 5) among older adults, with the effect slightly varying according to the type of social engagement. Importantly, this inverse association persisted across income levels, suggesting the benefits of employment extend beyond financial gains to include enhanced self-identity, social integration, and personal control, as supported by previous research [44]. These results align with prior studies showing that employment positively influences mental health in later life, even after controlling for physical health [45,46]. In Korea’s case, where social interactions tend to be more formal due to the influence of Confucian traditions, individuals with limited participation in social gatherings or leisure activities may face restricted social connectivity [47]. For these older adults, employment may serve as a compensatory mechanism by offering a structured environment that ensures a minimum level of social interaction [48].

However, policy proposals aimed at promoting employment among the elderly should be approached with caution. As demonstrated in previous results, the impact of employment can differ significantly depending on the context, and overly simplistic interpretations may be misleading. Some studies have reported that older adults engaged in physically demanding or low-quality jobs often experience poorer physical and mental health outcomes compared to those who retire voluntarily [49,50]. Korea has one of the highest elderly poverty rates in the world, and employment after retirement is likely to be a necessity rather than an individual choice [51]. A study of Korean older adults reported that an appropriate amount of working hours (<35h per week) and social engagement minimized the risk of depressive symptoms [28]. The South Korean government has implemented the Senior Employment Support Program since 2004, offering part-time work opportunities (15 hours per week) to older adults [52]. Participants who engaged in education-related work reported the highest scores in the subjective health status, self-esteem, and self-efficacy areas of quality of life compared to other types, and the longer the participation period, the greater the improvement in overall quality of life [53]. It should be noted, however, that employment in this study was measured as a binary variable (employed vs. not employed) and therefore does not reflect differences in job quality or working conditions that may influence its inverse association with mental health. In contexts where continued labor market participation is driven by economic necessity, intervention strategies must be tailored to individuals’ physical capacities and incorporate measures to minimize occupational health risks within their living environments [54].

Despite the strength of our study in that it used representative survey data from Korea, it also has several limitations. First, the use of cross-sectional data inherently limits the study design and precludes causal inference. Because the temporal sequence among social engagement, employment, and depressive symptoms cannot be established, reverse causality remains a plausible alternative explanation. Future longitudinal studies are needed to establish the temporal sequence and clarify causal pathways. Second, depressive symptoms were measured using self-reported data, which may be subject to response bias. Third, while the KCHS provides comprehensive population-based data, it may not adequately capture older adults with limited social engagement who are less likely to participate in community-based surveys. Fourth, the employment status variable in our study did not distinguish between different types of employment (e.g., full-time, part-time, self-employed) or the quality of employment, which could have varying impacts on mental health outcomes. Finally, despite controlling for a range of socioeconomic variables, there might be unmeasured confounding variables that could influence the relationship between social engagement and depressive symptoms.

## 5. Conclusion

Our findings demonstrated that employed older adults may be generally less vulnerable to depressive symptoms related to social engagement compared with their unemployed counterparts. The finding that employment reduces the risk of depressive symptoms in older adults was evident not only in high-income groups but also in low- and middle-income groups. These results have important implications for public health policies and interventions targeting depression prevention in aging populations. Further study is needed to determine how employment status is associated with mental health in socially isolated older adults to demonstrate causality.

## Supporting information

S1 Fig Association between frequency of informal social engagement and depressive symptoms. (PDF)

S2 Fig Association between social engagement (≥1/month vs < 1/month) and depressive symptoms by employment status. (PDF)

S1 Table Adjusted odds ratio (aOR) with 95% confidence interval (CI) for social engagement and depressive symptoms according to income and employment status. (PDF)

S2 Table Association between social engagement and depressive symptoms stratified by young-old (aged 65–74) and old-old (aged 75 and above). (PDF)

S3 Table Association between social engagement and depressive symptoms stratified by earlier period (2017, 2019) and recent period (2023). (PDF)

S4 Table Association between social engagement and depressive symptoms using a 10-point cutoff point. (PDF)
