# Supplementary material for: Social engagement and depressive symptoms in Korean older adults: The potential moderating role of employment status
Source: PLoS One. 2026 Mar 5;21(3):e0342299. doi: 10.1371/journal.pone.0342299 (PMC12962508; doi:10.1371/journal.pone.0342299)
Supplement: S1 Fig — (PDF) [file pone.0342299.s001.pdf]

Informal type of engagement	OR	95% CI	P for trend
**Contact with relative**			**<0.0001**
1-3/month vs <1/month	0.736	0.696-0.778	
≥1/week vs <1/month	0.675	0.642-0.710	
**Contact with neighbor**			**<0.0001**
1-3/month vs <1/month	0.742	0.698-0.789	
≥1/week vs <1/month	0.635	0.608-0.662	
**Contact with friend**			**<0.0001**
1-3/month vs <1/month	0.644	0.612-0.676	
≥1/week vs <1/month	0.585	0.562-0.609	

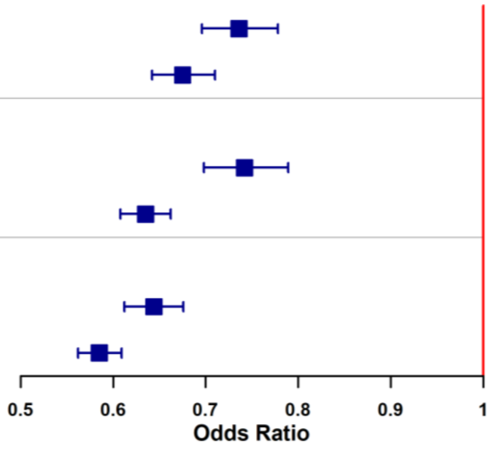

S1 Fig. Association between frequency of informal social engagement and depressive symptoms

Abbreviation: aOR, adjusted odds ratio; CI, confidence interval

Note: OR<1 indicates lower odds of depressive symptoms. Reference group is <1/month contact. Adjusted for age group, sex, marital status, education, living alone, household income, employment status, residence area, diabetes, hypertension, survey year, current smoking, current drinking, moderate-intensity physical activity.
