# Supplementary material for: Social engagement and depressive symptoms in Korean older adults: The potential moderating role of employment status
Source: PLoS One. 2026 Mar 5;21(3):e0342299. doi: 10.1371/journal.pone.0342299 (PMC12962508; doi:10.1371/journal.pone.0342299)
Supplement: S2 Fig — (PDF) [file pone.0342299.s002.pdf]

Employment Status	OR	95% CI
**Employed**		
Contact with relative	1.405	1.279-1.549
Contact with neighbor	1.376	1.261-1.501
Contact with friend	1.367	1.271-1.471
Religious activity	1.118	1.04-1.201
Social gatherings	1.280	1.199-1.366
Leisure/recreational activities	1.322	1.196-1.462
Charity/volunteer activities	1.123	0.968-1.302
**Unemployed**		
Contact with relative	1.444	1.367-1.525
Contact with neighbor	1.533	1.463-1.605
Contact with friend	1.71	1.638-1.785
Religious activity	1.222	1.169-1.278
Social gatherings	1.666	1.593-1.742
Leisure/recreational activities	1.53	1.431-1.636
Charity/volunteer activities	1.663	1.47-1.881

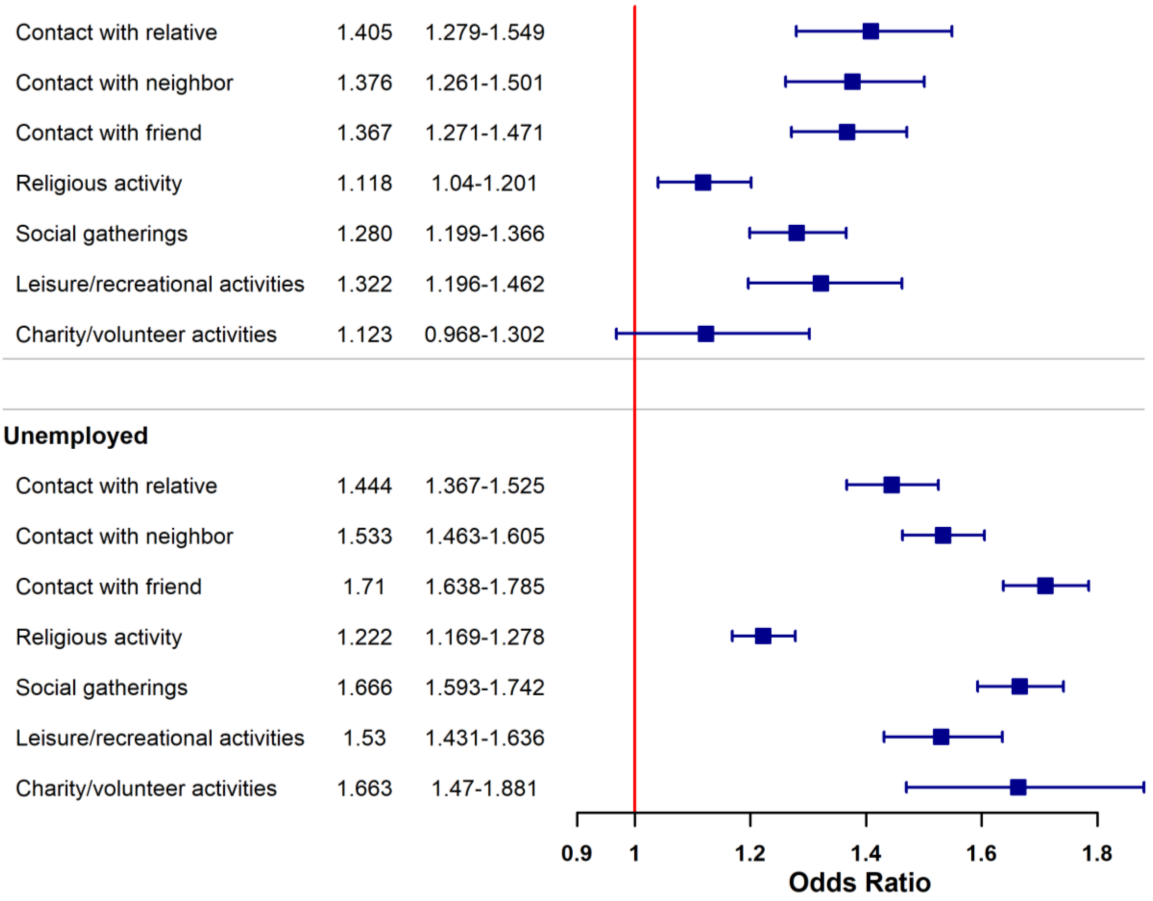

S1 Fig. Association between social engagement (≥1/month vs <1/month) and depressive symptoms by employment status

Abbreviation: aOR, adjusted odds ratio; CI, confidence interval

Note: Odds ratios and 95% confidence intervals from multivariable logistic regression models. Reference group: <1/month contact or participation. Models Adjusted for age group, sex, marital status, education, living alone, household income, employment status, residence area, diabetes, hypertension, survey year, current smoking, current drinking, moderate-intensity physical activity. OR>1 indicates higher odds of depressive symptoms.
