# Supplementary material for: Social engagement and depressive symptoms in Korean older adults: The potential moderating role of employment status
Source: PLoS One. 2026 Mar 5;21(3):e0342299. doi: 10.1371/journal.pone.0342299 (PMC12962508; doi:10.1371/journal.pone.0342299)
Supplement: S1 Table — (PDF) [file pone.0342299.s003.pdf]

S1 Table. Adjusted odds ratios (aOR) with 95% confidence intervals (CI) for social engagement and depressive symptoms according to income and employment status

Types of social engagement	aOR (95% CI)					
	Employment			Unemployment		
	Income low	Income mid	Income high	Income low	Income mid	Income high
Contact with relative (< 1 / month)	1.47(1.30,1.67)*	1.34(1.17,1.55)*	1.44(1.20,1.74)*	1.49(1.39,1.61)*	1.31(1.19,1.43)*	1.54(1.36,1.75)*
Contact with neighbor (< 1 / month)	1.41(1.24,1.61)*	1.44(1.28,1.62)*	1.28(1.11,1.48)*	1.50(1.40,1.60)*	1.50(1.40,1.62)*	1.61(1.46,1.78)*
Contact with friend (< 1 / month)	1.28(1.17,1.40)*	1.44(1.30,1.60)*	1.36(1.16,1.59)*	1.60(1.51,1.70)*	1.80(1.68,1.93)*	1.76(1.59,1.95)*
Religious activity (< 1 / month)	1.05(0.96,1.15)	1.16(1.05,1.29)*	1.12(0.97,1.29)	1.19(1.12,1.27)*	1.18(1.10,1.27)*	1.37(1.24,1.51)*
Social gatherings (< 1 / month)	1.32(1.21,1.44)*	1.37(1.25,1.50)*	1.08(0.94,1.24)	1.61(1.51,1.71)*	1.69(1.57,1.82)*	1.70(1.54,1.88)*
Leisure/recreational activities (< 1 / month)	1.30(1.12,1.50)*	1.46(1.27,1.68)*	1.17(0.99,1.38)	1.71(1.52,1.91)*	1.38(1.25,1.52)*	1.60(1.42,1.81)*
Charity/volunteer activities (< 1 / month)	1.16(0.91,1.49)	1.27(1.04,1.55)*	0.97(0.75,1.25)	2.10(1.69,2.62)*	1.58(1.33,1.89)*	1.49(1.19,1.88)*

Abbreviation: aOR, adjusted odds ratio; CI, confidence interval

Adjusted for age group, sex, marital status, education, living alone, household income, employment status, residence area, diabetes, hypertension, survey year, current smoking, current drinking, moderate-intensity physical activity.

*p<0
