# Supplementary material for: Social engagement and depressive symptoms in Korean older adults: The potential moderating role of employment status
Source: PLoS One. 2026 Mar 5;21(3):e0342299. doi: 10.1371/journal.pone.0342299 (PMC12962508; doi:10.1371/journal.pone.0342299)
Supplement: S2 Table — (PDF) [file pone.0342299.s004.pdf]

S2 Table. Association between social engagement and depressive symptoms stratified by young-old (aged 65-74) and old-old (aged 75 and above).

		aOR (95% CI)		Interaction P value
**Variables**	Categories	Young-old	Old-old	
Contact with relative	≥1 / month	1	1	0.0012
	< 1 / month	1.53(1.43-1.63)*	1.33(1.24-1.42)*	
Contact with neighbor	≥1 / month	1	1	<0.0001
	< 1 / month	1.47(1.39-1.56)*	1.53(1.45-1.62)*	
Contact with friend	≥1 / month	1	1	<0.0001
	< 1 / month	1.60(1.52-1.69)*	1.69(1.61-1.78)*	
Religious activity	≥1 / month	1	1	0.36
	< 1 / month	1.17(1.10-1.23)*	1.24(1.18-1.31)*	
Social gatherings	≥1 / month	1	1	0.49
	< 1 / month	1.56(1.48-1.64)*	1.59(1.51-1.67)*	
Leisure/recreational activities	≥1 / month	1	1	0.0003
	< 1 / month	1.41(1.32-1.52)*	1.72(1.56-1.89)*	
Charity/volunteer activities	≥1 / month	1	1	0.74
	< 1 / month	1.46(1.30-1.64)*	1.50(1.26-1.78)	

Abbreviation: aOR, adjusted odds ratio; CI, confidence interval

Adjusted for age group, sex, marital status, education, living alone, household income, employment status, residence area, diabetes, hypertension, survey year, current smoking, current drinking, moderate-intensity physical activity.

*p<0.05
