# Supplementary material for: Social engagement and depressive symptoms in Korean older adults: The potential moderating role of employment status
Source: PLoS One. 2026 Mar 5;21(3):e0342299. doi: 10.1371/journal.pone.0342299 (PMC12962508; doi:10.1371/journal.pone.0342299)
Supplement: S3 Table — (PDF) [file pone.0342299.s005.pdf]

S3 Table. Association between social engagement and depressive symptoms stratified by earlier period (2017, 2019) and recent period (2023)

		aOR (95% CI)		**Interaction P value**
**Variables**	Categories	Earlier period (2017,2019)	Recent period (2023)	
Contact with relative	≥1 / month	1	1	0.02
	< 1 / month	1.38(1.29-1.47)*	1.53(1.43-1.64)*	
Contact with neighbor	≥1 / month	1	1	0.01
	< 1 / month	1.45(1.37-1.53)*	1.58(1.49-1.66)*	
Contact with friend	≥1 / month	1	1	0.67
	< 1 / month	1.64(1.56-1.72)*	1.66(1.58-1.75)*	
Religious activity	≥1 / month	1	1	0.43
	< 1 / month	1.17(1.12-1.24)*	1.24(1.17-1.30)*	
Social gatherings	≥1 / month	1	1	0.33
	< 1 / month	1.55(1.48-1.63)*	1.60(1.52-1.68)*	
Leisure/recreational activities	≥1 / month	1	1	0.89
	< 1 / month	1.49(1.38-1.61)*	1.52(1.41-1.63)	
Charity/volunteer activities	≥1 / month	1	1	0.77
	< 1 / month	1.44(1.26-1.65)*	1.50(1.32-1.71)*	

Abbreviation: aOR, adjusted odds ratio; CI, confidence interval

Adjusted for age group, sex, marital status, education, living alone, household income, employment status, residence area, diabetes, hypertension, survey year, current smoking, current drinking, moderate-intensity physical activity.

*p<0.05
