# Supplementary material for: Social engagement and depressive symptoms in Korean older adults: The potential moderating role of employment status
Source: PLoS One. 2026 Mar 5;21(3):e0342299. doi: 10.1371/journal.pone.0342299 (PMC12962508; doi:10.1371/journal.pone.0342299)
Supplement: S4 Table — (PDF) [file pone.0342299.s006.pdf]

S4 Table. Association between social engagement and depressive symptoms using a 10-point cutoff point

Variables	Categories	aOR (95% CI)
Contact with relative	≥1 / month	1
	< 1 / month	1.62(1.50-1.76)*
Contact with neighbor	≥1 / month	1
	< 1 / month	1.86(1.74-2.00)*
Contact with friend	≥1 / month	1
	< 1 / month	1.99(1.87-2.13)*
Religious activity	≥1 / month	1
	< 1 / month	1.31(1.22-1.41)*
Social gatherings	≥1 / month	1
	< 1 / month	2.09(1.94-2.25)*
Leisure/recreational activities	≥1 / month	1
	< 1 / month	2.12(1.86-2.42)*
Charity/volunteer activities	≥1 / month	1
	< 1 / month	2.15(1.71-2.71)*

Abbreviation: aOR, adjusted odds ratio; CI, confidence interval

Adjusted for age group, sex, marital status, education, living alone, household income, employment status, residence area, diabetes, hypertension, survey year, current smoking, current drinking, moderate-intensity physical activity.

*$p<0.05$
